# *Theileria*’s Strategies and Effector Mechanisms for Host Cell Transformation: From Invasion to Immortalization

**DOI:** 10.3389/fcell.2021.662805

**Published:** 2021-04-20

**Authors:** Kerry Woods, Carmen Perry, Francis Brühlmann, Philipp Olias

**Affiliations:** Institute of Animal Pathology, Vetsuisse Faculty, University of Bern, Bern, Switzerland

**Keywords:** *Theileria*, Apicomplexa, invasion, annulate lamellae, microtubule, transformation, *Toxoplasma*, *Plasmodium*

## Abstract

One of the first events that follows invasion of leukocytes by *Theileria* sporozoites is the destruction of the surrounding host cell membrane and the rapid association of the intracellular parasite with host microtubules. This is essential for the parasite to establish its niche within the cytoplasm of the invaded leukocyte and sets *Theileria* spp. apart from other members of the apicomplexan phylum such as *Toxoplasma gondii* and *Plasmodium* spp., which reside within the confines of a host-derived parasitophorous vacuole. After establishing infection, transforming *Theileria* species (*T. annulata*, *T. parva*) significantly rewire the signaling pathways of their bovine host cell, causing continual proliferation and resistance to ligand-induced apoptosis, and conferring invasive properties on the parasitized cell. Having transformed its target cell, *Theileria* hijacks the mitotic machinery to ensure its persistence in the cytoplasm of the dividing cell. Some of the parasite and bovine proteins involved in parasite-microtubule interactions have been fairly well characterized, and the schizont expresses at least two proteins on its membrane that contain conserved microtubule binding motifs. *Theileria*-encoded proteins have been shown to be translocated to the host cell cytoplasm and nucleus where they have the potential to directly modify signaling pathways and host gene expression. However, little is known about their mode of action, and even less about how these proteins are secreted by the parasite and trafficked to their target location. In this review we explore the strategies employed by *Theileria* to transform leukocytes, from sporozoite invasion until immortalization of the host cell has been established. We discuss the recent description of nuclear pore-like complexes that accumulate on membranes close to the schizont surface. Finally, we consider putative mechanisms of protein and nutrient exchange that might occur between the parasite and the host. We focus in particular on differences and similarities with recent discoveries in *T. gondii* and *Plasmodium* species.

## Introduction

*Theileria* is a genus of tick-borne parasites and, together with *Babesia* spp., forms the order of Piroplasmida within the phylum of Apicomplexa. Apicomplexan parasites are important zoonotic and human pathogens and include members such as *Plasmodium* spp., the causative agents of malaria and responsible for more than 400,000 deaths per year (World Malaria Report, 2020); *Toxoplasma gondii*, arguably the most successful parasite worldwide capable of infecting all warm blooded animals and estimated to infect up to 30% of the human population (Aguirre et al., 2019); *Babesia* spp., for which humans are accidental hosts (Yabsley and Shock, 2013); and *Theileria* spp. that cause devastating diseases of cattle and result in huge economic losses in several countries of the Global South (Morrison, 2015). Due to their large impact on human and animal health, apicomplexan parasites have been the subject of extensive research efforts which have uncovered fascinating mechanisms of host cell manipulation, ranging from metabolic reprogramming to hijacking of host gene expression (recently reviewed by Villares et al., 2020). Among the most striking alterations of host cell phenotype are induced by the pathogenic *Theileria* species *T. annulata* and *T. parva*. Upon infection of bovine leukocytes, these parasites re-program cell signaling pathways to such an extent that they induce cellular transformation, conferring a proliferative phenotype and immortality on infected leukocytes (Dobbelaere and Rottenberg, 2003; Tretina et al., 2015). *Theileria*-infected leukocytes thus share many key hallmarks with cancer, and the clonal expansion of infected leukocytes and the associated cytokine storm have been implicated in pathogenesis (Glass et al., 2012). In infected cattle this causes East Coast Fever (*T. parva*) and Tropical Theileriosis (*T. annulata*), comprising symptoms including fever, anemia and enlarged lymph nodes. In many cases infection results in the death of the animal. The *Theileria* genus also comprises the so-called “non-transforming” species *T. orientalis*, which does not induce proliferation in the invaded blood cells and consequently does not cause lymphoproliferative diseases in livestock, although infection can still be lethal due to severe anemia (Watts et al., 2016).

Infective sporozoites are transmitted by ticks and invade white blood cells of cattle and other small ruminants, with *T. annulata* infecting bovine macrophages and B cells, and *T. parva* infecting bovine T cells and B cells (Spooner et al., 1989). Following invasion, the sporozoite develops into a multinucleated syncytium called a macroschizont, and proliferation of the parasitized leukocyte is triggered within three days of infection. Rather than undergoing multiple rounds of egress and reinvasion like *T. gondii*, *Theileria* schizonts interact closely with the mitotic machinery of the host cell and are divided to both daughter cells following cytokinesis, thus leading to clonal expansion of the schizonts together with the infected leukocytes. *Theileria* infection is associated with extensive changes in host cell kinase activity, and a significant increase in total protein phosphorylation in infected cells has been reported (ole-MoiYoi et al., 1993). *Theileria* schizonts take control of multiple signaling pathways in the host, leading to changes in gene expression that favor parasite survival and spread of the infected cell. One important example is the constitutive activation of the NF-κB pathway that is essential for protecting the *Theileria*-transformed leukocyte from apoptosis (Heussler et al., 2002). Another example is the AP-1-driven expression of metalloprotease-9 (MMP9) that confers an invasive phenotype on the infected host (Baylis et al., 1995). Intriguingly, survival and the maintenance of the transformed phenotype in *Theileria*-infected cells depends on the presence of a viable parasite, suggesting that transformation is driven by changes in gene expression and cell signaling pathways rather than mutations or alterations at the DNA level (Dobbelaere and Rottenberg, 2003). Although the extensive changes in host cell signaling pathways induced by *Theileria* infection have been relatively well studied (see reviews Woods K. et al., 2013; Cheeseman and Weitzman, 2015; Tretina et al., 2015), few parasite effector proteins have been characterized in detail, and even fewer have been linked convincingly to regulation of the transformed host cell phenotype.

In this review, we focus on host-parasite interactions in *Theileria*-infected leukocytes, in particular *Theileria* proteins that interact with host cell proteins at the schizont membrane, and those that are secreted into the host cytoplasm or nucleus where they have the potential to modify the host phenotype. We discuss the recent description of host-derived pore-containing membranes that align close to the schizont membrane, and we speculate on some of the shared and unique elements of *Theileria* protein export as compared to other apicomplexans. In addition to reviewing published work that addresses important elements of the *Theileria*-leukocyte interaction, we sought to identify some key open questions that will hopefully motivate further study into *Theileria* biology.

## Invasion in Any Orientation

Like most other apicomplexan parasites, *Theileria* have a complex life cycle (reviewed in Jalovecka et al., 2018). The invasion process of tick-borne *Theileria* sporozoites into their mammalian target cells differs strikingly from that of other apicomplexans such as *Toxoplasma* and *Plasmodium* and these differences are reflected in the morphology of the invasive sporozoites. *Theileria* sporozoites are approximately spherical, 0.75 – 1.5 μm in diameter, have a single nucleus occupying the basal region of the cell, and are covered by a 20 – 25 nm surface coat which is trypsin-sensitive (Fawcett et al., 1982; Shaw, 1991). Their cytoplasmic hemisphere contains a single mitochondrion without cristae, numerous free ribosomes, and dispersed secretory organelles named microspheres (equivalent to dense granules in *T. gondii* and *Plasmodium* spp.). The apical tip of the sporozoite is defined by few rhoptries attaching to the cell membrane via a peg-like structure similar to a polar ring, but otherwise lacks typical structures of a large apical complex such as a conoid (as do all piroplasms; class Aconoida), a subpellicular microtubule basket, and apical micronemes. Electron microscopy studies indicate that *Theileria* sporozoites are surrounded only by a plasma membrane and do not possess an inner membrane complex (Stagg et al., 1981; Jura et al., 1983; Shaw, 1991). Thus, *Theileria*‘s sporozoite structure differs greatly from the larger (4 – 14 μm) and elongated sporozoites of *Toxoplasma* and *Plasmodium* (Speer et al., 1995; Frischknecht and Matuschewski, 2017). The lack of specialized secretory micronemes in the apical region of sporozoites, which in other apicomplexans release proteins essential for parasite gliding motility and are required for host cell attachment and invasion (Frénal et al., 2017), further reflects differences in the invasion process.

In contrast to all other apicomplexans, including the closely related *Babesia* spp. (Jalovecka et al., 2018), *Theileria* sporozoites are non-motile and their contact with a host cell happens by chance (Shaw, 1991). This initial attachment is irreversible and of an essentially passive nature, though inhibitable by proteases, suggesting the involvement of specific host-parasite surface molecule interactions. Invasion occurs within three minutes after attachment and the parasite is established inside the host cell cytosol within 15 min. Unlike other apicomplexan zoites, *Theileria* sporozoites do not reorientate for invasion and can enter the host cell in any direction. During this process parasite and host cell membranes are in close contact, and entry seems to occur rather passively by a “circumferential zippering” of the two membranes. As no involvement of the actin cytoskeleton of the parasite has been shown and no actin accumulation or pseudopodia formation occurs at the leukocyte surface, it is supposed that this process of zippering might be sufficient in itself to lead to internalization of the sporozoite (Fawcett et al., 1982). Invasion can be blocked by pre-treating the host lymphocytes with cytoskeleton-disrupting reagents (Shaw, 1991), though it is not known whether this inhibition is due to a reduction in sporozoite binding caused by altered surface receptor distribution on the host cell, or whether the invasion itself cannot take place. Though not fully understood, the invasion process of *Theileria* sporozoites is starkly different to, for example, the invasion of *Toxoplasma* tachyzoites, which is actively driven by parasite motility and involves the formation of moving junctions, complexes of parasite proteins interacting with the host cell surface to support penetration of the host cell (Frénal et al., 2017). During the internalization and zippering process, parts of the *Theileria* sporozoite surface coat are shed (Fawcett et al., 1984; Webster et al., 1985) including the *T. parva* surface protein p67 (TP03_0287) (Shaw, 2002). This suggests that successful invasion involves proteolytic processing of parasite or host surface molecules, which is supported by the fact that entry can be blocked by protease inhibitors. In *Theileria* sporozoites, microspheres and rhoptries are discharged approximately 15 min post-invasion and seem to be controlled by a timed mechanism starting at the moment of attachment, as discharge occurs still during invasion if the invasion process is slowed down with cytochalasin D (Shaw, 1991).

Molecularly, little is known about the invasion process and the host and parasite molecules that are involved. For *T. parva*, two sporozoite surface molecules have been identified which seem to play a role in invasion, namely p67 (Dobbelaere et al., 1985; Iams et al., 1990a) and PIM (Polymorphic immunodominant molecule; TP04_0051; the orthologs protein is named TaSP in *T. annulata*; TA17315) (Toye et al., 1991). For *T. annulata*, the major sporozoite surface protein is SPAG-1 (TA03755), which shares considerable sequence homology with p67. Antibodies against p67 and SPAG-1 have been shown to block sporozoite entry into the host cell (Williamson et al., 1989; Musoke, 1992) and immunization with either antigen provides some cross-species protection (Hall et al., 2000). Native and recombinant p67 competitively and reversibly blocks sporozoite binding, indicating that p67 interacts with the host cell, though in a rather weak manner (Shaw et al., 1995). Because attachment of the sporozoite to the host cell is quite strong and most likely irreversible, it is supposed that this attachment is mediated via numerous interactions resulting from the high density of p67 distributed on the sporozoite surface (Shaw, 2002). In contrast to *Theileria*, the molecular components of the invasion process of *Toxoplasma* and *Plasmodium* zoites have been well studied. Apical membrane antigen 1 (AMA1) is a secreted microneme protein conserved across the Apicomplexa that, by interacting with RON2, plays a critical role in the formation of the moving junction and host cell invasion (Hehl et al., 2000; Bargieri et al., 2013). Other well-characterized proteins required for host invasion are the *Plasmodium* thrombospondin-related anonymous protein (TRAP) and the *T. gondii* homolog microneme protein 2 (MIC2) (Sultan et al., 1997; Huynh and Carruthers, 2006). Although *Theileria* sporozoites are non-motile and their invasion process is considered rather passive, *Theileria* parasites do possess homologs of TRAP (TA07755) and AMA1 (TA02980), neither of which have been studied in *Theileria*.

While some hepatocyte receptors involved in *Plasmodium* sporozoite invasion are relatively well known (for example CD81 and SR-B1, reviewed in Dundas et al., 2019), it is not known which host cell molecules *Theileria* sporozoites interact with. It is likely that sporozoite surface markers recognize very specific host molecules, allowing them to selectively invade only a restricted population of host cells. It has been shown that antibodies against MHC class l and β-2 microglobulin block *T. parva* sporozoite invasion and that these molecules are an essential component of the host receptor involved in sporozoite-host binding (Shaw, 1991, Shaw et al., 1995). *T. annulata*, on the other hand, invades cells expressing MHC class ll molecules (Spooner et al., 1989), though no studies have been performed to show that MHC class ll molecules are directly involved in invasion. It is likely that the attachment and invasion process includes interactions with other surface molecules as well, as interaction with MHC class l or ll molecules would not explain the high selectivity in invasion.

## Establishing a Niche in the Host Cytoplasm

After invasion, the sporozoite is first encapsulated in a membrane of host cell origin, called the parasitophorous vacuole (PV). However, in contrast to many other apicomplexan parasites (e.g., *Toxoplasma* and *Plasmodium*), *Theileria*, as well as *Babesia*, rapidly escape this host membrane after invasion and reside directly in the host cell cytosol (Shaw, 1991; Repnik et al., 2015). The separation of host and parasite membranes in *Theileria* occurs simultaneously with rhoptry and microsphere discharge and starts at the regions where rhoptries are located (Shaw, 1991). Unlike in other apicomplexans this seems not to be calcium-dependent (Shaw, 1991, 1997), and the escape from the host cell membrane is not dependent on the acidification of the parasite-containing vacuole as is the case for other intracytoplasmic pathogens such as *Listeria* (Li et al., 2017) and *Trypanosoma* (Batista et al., 2020). The first visible reaction of the host cell to the presence of the parasite is the arrangement of orderly microtubules (MTs) which associate with the parasite surface shortly after PV membrane (PVM) lysis and converge toward the centriole-Golgi region (Shaw, 1991).

## Clasping Microtubules at the Schizont Surface

**FIGURE 1:**
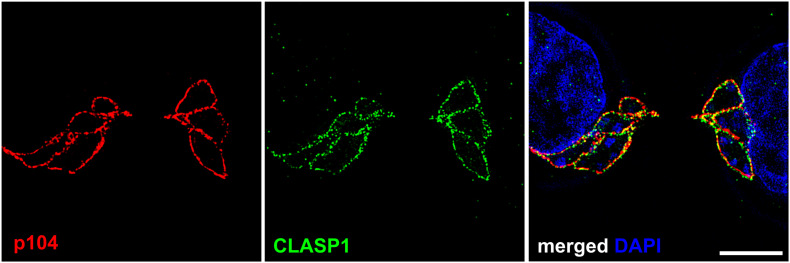
Punctate localization of *Theileria annulata* p104 and bovine CLASP1 on the schizont membrane. Structured illumination microscopy (SIM) was performed on *T. annulata* infected macrophages (TaC12) with anti-p104 (red) and anti-CLASP1 (green) antibodies. *Z*-stack images were taken at 12.5 nm intervals, one *z*-stack is shown. Scale bar is 5 μm.

*Theileria* schizonts have evolved a unique mechanism to facilitate their expansion in an infected cow. Upon development into a schizont, *Theileria* triggers dramatic changes in the phenotype of the infected cell, inducing uncontrolled proliferation that leads to the clonal expansion of infected leukocytes. Rather than egress and reinvade new host cells, *Theileria* hijacks the mitotic machinery of the host cell and aligns itself with the mitotic spindle to ensure its segregation to both daughter cells following cytokinesis of the transformed cell (von Schubert et al., 2010). The close association of *Theileria* with host MTs is observed very soon following PVM rupture and is maintained throughout the schizont stage. Division of the host cell into two daughter cells with the parasite being distributed equally between them happens as early as three days after infection (Hulliger et al., 1964; Stagg et al., 1981). MTs bind closely to the surface of the schizont and the parasite “incorporates” itself within the host central spindle during cytokinesis, ensuring its distribution to both throughout the cell cycle (Figure 1; Huber et al., 2017).

Live imaging of *Theileria*-infected cells stably expressing RFP-tubulin revealed that mitotic MTs aligned with the schizont surface are stable, unlike cytoplasmic MTs which are highly dynamic (von Schubert et al., 2010). Over the last decade a number of host proteins, including kinases and MT-associated proteins (MAPs) involved in regulating MT dynamics, have been found to localize close to the schizont membrane where they likely promote the stabilization of MTs. Von Schubert and colleagues described the biphasic association of the host mitotic kinase polo-like kinase 1 (Plk1) with the schizont surface (von Schubert et al., 2010). Plk1 is a mitotic kinase, expressed at high levels during G2 phase and mitosis, and degraded at the end of mitosis. Plk1 has a large array of interaction partners and phosphorylation targets, and by phosphorylating different targets, it controls numerous elements of the cytokinesis process including contractile ring formation and cleavage furrow ingression (Petronczki et al., 2008). Plk1 binding to the schizont is mediated via its Polo-box domain, is most prominent during G2 phase and anaphase, and is conspicuously absent during prometaphase and metaphase when Cdk1 activity is highest. Most importantly, inhibition of Plk1 activity with potent anti-Plk1 inhibitors prevented the binding of the schizont with the stable MTs of the central spindle. When the schizont-central spindle interaction was prevented by Plk1 inhibition at the onset of anaphase (thus still allowing spindle formation and cleavage furrow ingression), and at the same time the association of astral MTs with the parasite was blocked by depolymerization of MTs with nocodazole, the parasite was no longer properly segregated during cell division, often resulting in parasites being sequestered on one side of the cleavage furrow (von Schubert et al., 2010). This clearly demonstrates that the proper interaction between the parasite and MTs is crucial for the persistence of the schizont in the cytoplasm of the host, and that this interaction is at least in part mediated by Plk1 activity.

One parasite-encoded molecule that is involved in the parasite-MT interaction is p104 (TA08425), an immunodominant molecule that is expressed at the surface of the *T. annulata* schizont (Woods K. L. et al., 2013). p104 possesses a predicted signal peptide and cleavage site as well as a predicted GPI anchor sequence, although the functionality of the GPI anchor sequence has not been confirmed. While the N-terminal portion of p104 is structured and contains repeated FAINT (frequently associated in *Theileria*) domains that are unique to *Theileria*, the C-terminal half of the protein is highly disordered and contains a conserved End Binding Protein 1 (EB1)-binding SxIP motif. EB1 is a host cell MT-plus end binding protein that localizes to the plus ends of growing MTs where, by interacting transiently and dynamically with hundreds of different proteins, it plays a key role in regulating the dynamics of MTs (Gouveia and Akhmanova, 2010). *T. annulata* taps into the MT network by hijacking host EB1, a key regulator of MT dynamics, and EB1 associates with the schizont surface by a SxIP-motif dependent interaction with p104. Thus it seems that MTs are directed to the parasite surface via the p104-EB1 interaction (Woods K. L. et al., 2013).

The presence of EB1-binding motifs at the schizont membrane are not alone sufficient to explain the capture and subsequent stabilization of MTs along the surface of the parasite, and in this context the host MT-stabilizing protein CLASP1 was found to coat the entire schizont throughout the cell cycle (Figure 1; Huber et al., 2017). Even following isolation of the schizont with a method that requires depolymerization of MTs, bovine CLASP1 remains firmly bound to the schizont surface. CLASP1 functions to stabilize MTs by facilitating MT rescue and limiting catastrophe by recruiting tubulin dimers (Al-Bassam et al., 2010; Leano and Slep, 2019). It was previously shown that isolated schizonts can facilitate MT binding and polymerization at their surface (Kuehni-Boghenbor et al., 2012), and it is likely that CLASP1 plays a role in this. CLASP1, a known EB1-binding protein, contains two SxIP motifs which presumably also contribute to the interaction of host EB1 with the parasite. The CLASP1 SxIP-motifs are not required for parasite localization, because the short C-terminal kinetochore-binding domain was found to be sufficient and necessary for the interaction with the schizont membrane (Huber et al., 2017). Co-immunoprecipitation and proximity ligation experiments confirmed that CLASP1 interacts with both p104 and EB1 at the schizont surface. Western blotting and mass spectrometry analysis revealed that p104 is heavily phosphorylated in a cell cycle dependent manner, with both endogenous and ectopically expressed p104 being hyper-phosphorylated during mitosis in a manner at least in part regulated by host Cdk1 activity (Woods K. L. et al., 2013; Wiens et al., 2014). EB1 binding to the schizont, like that of Plk1, correlates inversely to host Cdk1 activity and the hyper-phoshorylation of *T. annulata* p104. Interestingly, host Cdkl1 has recently been found to partially co-localize with and phosphorylate the parasite surface molecule TaSP (*T. annulata* surface protein, TA17315), and it was postulated that phosphorylated TaSP might act as a docking protein for other cell cycle regulatory proteins (Mackiewicz et al., 2020).

CLASP2 is a close homolog of CLASP1 and both proteins share largely overlapping functions and localization in mammalian cells (Mimori-Kiyosue et al., 2006). CLASP2 is also enriched at the schizont surface, but in a MT-dependent manner with a localization resembling that of tubulin (Huber et al., 2017). Knockdown of CLASP1 expression in *Theileria*-infected cells had no effect on schizont position, morphology or distribution during host cytokinesis, presumably due to the continued presence of CLASP2. Over-expression of the CLASP1 MT-binding domain did however have a striking dominant negative effect on infected cells with large, round parasites residing in enlarged host cells containing bundled MTs. While this experiment emphasized the importance of a properly functioning host MT network on *Theileria* morphology and structure, the MT-binding domain of CLASP1 also has a dominant negative effect on non-infected cells (Maiato et al., 2003), making it difficult to separate the dependencies of host and parasite on a functional MT network.

End Binding Protein 1, Plk1 and CLASP1 are not the only host cell proteins to be sequestered at the schizont. The striking localization of CLASP1 at the schizont surface meant that the kinetochore binding fragment of CLASP1 served as an effective bait for proximity ligation pull-down experiments to identify further host-parasite interactions. These experiments led to the identification of a number of host adaptor proteins that coat the schizont surface including CD2 associated protein (CD2AP), c-Cbl-interacting protein of 85 kDa (CIN85) and Arf-GAP with SHD domain ANK repeat and PH domain-containing protein 1 (ASAP1) (Huber et al., 2018). Adaptor proteins are proteins that contain several protein-binding domains that facilitate the formation of large signaling complexes (Flynn, 2001). CD2AP and CIN85 interact with numerous binding partners and have been implicated in diverse biological processes including vesicle trafficking, cytokinesis and cytoskeleton dynamics (reviewed in Dikic, 2002). It is tempting to speculate that some of these adaptor proteins may contribute to the generation of signaling complexes at the parasite surface, and thus contribute to the parasite-driven remodeling of the host. CD2AP and CIN85 are also recruited by *Toxoplasma* to the moving junction during invasion, and invasion-deficient *T. gondii* mutants with depleted rhoptry proteins RON2, RON4, and RON5 fail to recruit CD2AP/CIN85 (Guérin et al., 2017). However, it is unlikely that CD2AP and CIN85 play any role in *Theileria* invasion because these proteins were not detected at the parasite surface until development into a schizont had started (Huber et al., 2018).

Host CD2AP, CIN85 and ASAP1 were found to form a complex at the parasite surface together with host CLASP1 and EB1, *T. annulata* p104 and a novel *T. annulata* encoded protein named *T. annulata* proline rich microtubule and SH-domain interaction protein (TaMISHIP, TA20980, Huber et al., 2018). TaMISHIP is a protein that contains a signal peptide and predicted cleavage site, is highly disordered, and is unique to transforming species of *Theileria*. TaMISHIP partially co-localizes with p104 in sporozoites, presumably in rhoptry organelles, and upon schizont development TaMISHIP is found in a punctate pattern in close proximity to p104 on the parasite membrane (Huber et al., 2018). A *T. annulata* GPI anchored protein named GPI anchored schizont protein 34 (gp34; TA06510) was also found to be present in the TaMISHIP-protein complex at the schizont surface (Huber et al., 2018). Gp34, like TaMISHIP, covers the schizont surface in punctate foci (Xue et al., 2010; Huber et al., 2018). Upon over-expression in infected or non-infected cells, both TaMISHIP and gp34 translocate to the nucleus and induce a small but significant increase in binucleation. Although it is unlikely that the nuclear localization of over-expressed TaMISHIP or gp34 reflect the localization of the endogenous proteins, these experiments, combined with the fact that both TaMISHIP and gp34 are part of a complex including MT-regulators CLASP1 and EB1, indicate that both proteins might contribute to host-parasite interactions during host cell division (Xue et al., 2010). TaMISHIP encompasses two EB1-binding SxIP motifs, and live cell imaging with uninfected cells over-expressing GFP-TaMISHIP revealed that TaMISHIP tracks the plus ends of growing MTs (Huber et al., 2018). Mutation of the SxIP motif abolished plus end tracking, confirming that the interaction between TaMISHIP and EB1 is, like p104, mediated via the SxIP motif. TaMISHIP also contains three putative SH3-binding Px(P/A)xPR motifs which are predicted to mediate interactions with SH3 domains. Mutational studies combined with co-immunoprecipitation showed that the interaction between TaMISHIP and CD2AP, which contains three SH3 domains, is mediated via the SH3 domains of TaMISHIP (Huber et al., 2018).

While a number of host and parasite molecules that contribute to maintaining the tight association between the schizont and host MTs have been identified, many questions remain open. How the final stages of cytokinesis and abscission are regulated in infected cells is worthy of further investigation. *Theileria* synchronizes its cell cycle to that of its host by initiating DNA synthesis during host mitosis (Irvin et al., 1982; Wiens et al., 2014), but how these processes are controlled and coordinated – does host cell cycle progression drive parasite DNA synthesis, or vice versa? – remains unknown.

The host MT network is also rearranged and recruited to the *T. gondii* PVM. Differently to *Theileria*-infected cells, *T. gondii* infected cells rarely complete cytokinesis, and it has been proposed that the interactions between *T. gondii* and host MTs might suppress cell division and therefore provide a larger space in which the parasite can replicate (Walker et al., 2008). The rearrangement of host organelles in *T. gondii* infected cells is well documented. For example, the mitochondria and rough ER physically associate with the PV (Sinai et al., 1997), and host endolysosomes and Rab vesicles cluster round the PV and have been implicated in nutrient uptake (Coppens et al., 2006; Coppens and Romano, 2018). It seems likely that *T. gondii* rearranges host MT networks in order to facilitate the rearrangement of intracellular organelles to the benefit of the parasite. Although it has not been shown in *Theileria*-infected cells, a similar recruitment of host organelles such as the ER, golgi, mitochondria or endolysosomal vesicles is possible (Stagg et al., 1981).

## The Elusive Role of Porous Annulate Lamellae at the Host-Parasite Interface

**FIGURE 2:**
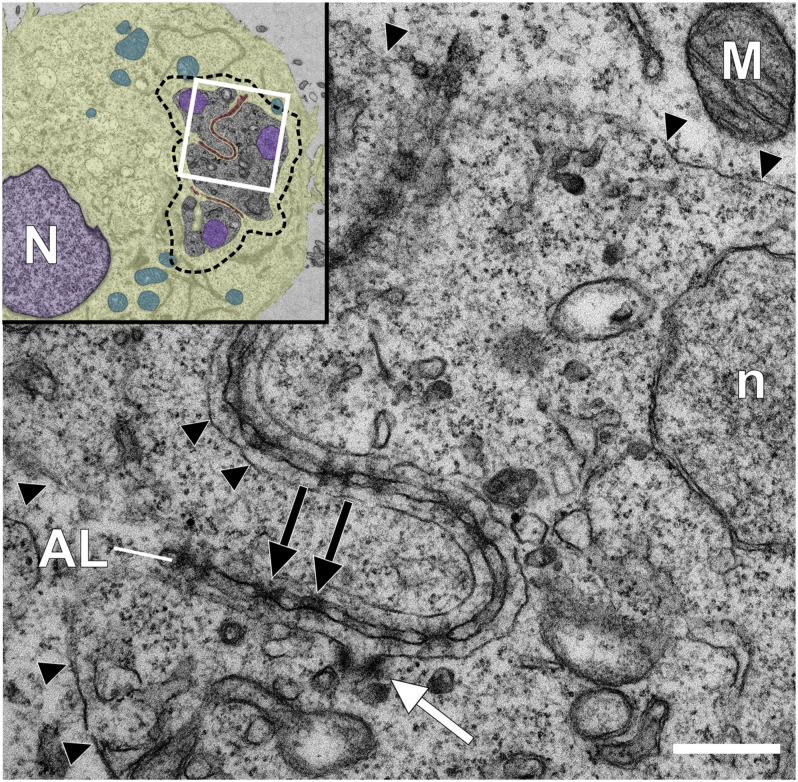
Transmission electron microscopy analysis of a *Theileria annulata* infected macrophage reveals annulate lamellae pore complexes close to the schizont surface. Glutaraldehyde-fixed and Epon-embedded TaC12 cells were analyzed using TEM. Annulate lamellae (AL) and AL pore complexes (ALPC, black arrows) are indicated. The parasite plasma membrane (PPM) is indicated with black arrow heads. The white arrow indicates a putative cytostome. Scale bar is 400 nm. The white box in the inset indicates the magnified area. Inset: overview of the infected cell. Host (N) and parasite (n) nuclei have been colored purple, the host cytoplasm has been colored yellow, host mitochondria are blue, and host derived AL are highlighted brown. The outline of the schizont is indicated with a dashed line.

Recent transmission electron microscopy (TEM) combined with high resolution fluorescent imaging revealed that *Theileria* schizonts are surrounded by porous, cytomembranous structures called annulate lamellae (AL) during host interphase (Figure 2; Huber et al., 2020). AL are poorly characterized cytoplasmic organelles composed of stacked membrane cisternae which often occur in rapidly growing cells such as embryonic cells, oocytes and tumor cells (Kessel, 1992). AL contain pores that are both structurally and biochemically similar to nuclear pore complexes (NPCs). In *Theileria*-infected leukocytes, these host-derived porous membranes align close to, but not touching, the schizont membrane, and were often detected in between the lobes of the schizont (Figure 2; Huber et al., 2020). The AL disperse as the cell enters mitosis with the same dynamics as the host nuclear envelope, reforming and realigning close to the schizont membrane upon completion of cytokinesis. Almost all known structural components of NPCs associate with AL pore complexes (ALPCs) at the parasite plasma membrane (PPM), along with components of nuclear trafficking machinery. Host cell proteins that accumulate at AL close to the parasite membrane include the small GTPase Ran, RanGTPase activating protein (RanGAP1), Ran binding protein (RanBP2) and importin (Huber et al., 2020). NLS-domain containing proteins destined for nuclear import bind to karyopherins such as importin and are trafficked into the nucleus upon the formation of a Ran GTPase gradient between the nucleus and the cytoplasm (D’Angelo and Hetzer, 2008). Although experimental evidence is lacking, a model by which secreted nuclear localization signal (NLS)-containing *Theileria* proteins are “picked up” at the PPM by host importin and trafficked into the nucleus of the host is very attractive and would be interesting to investigate further.

Whether *Theileria* schizonts depend on host cell nutrients or metabolites remains unexplored, although genomic analyses have suggested that, like other Apicomplexa, *Theileria* may have reduced metabolic capacity that could indicate a reliance on host cell metabolite-scavenging (Hayashida et al., 2012). If this is the case, the potential mechanism by which nutrients could be imported over the PPM is not known. The schizont membrane has a lobular shape and possesses a rather large membrane surface that is exposed to the host cell. Membrane contact sites between intracellular organelles have emerged in recent years as important hot spots for cellular signaling, lipid exchange and communication (Jain and Holthuis, 2017; Bohnert, 2020). While regions of direct contact between the schizont PPM and porous AL or other intracellular organelles have not been detected, cytostome-like structures are frequently observed embedded in the schizont membrane at points of close proximity to AL, raising the possibility of the direct transfer of lipids or metabolites between the parasite and host via these structures (Figure 2; Huber et al., 2020). Filamentous protrusions resembling nanotubes described from *Plasmodium* gametocytes (Rupp et al., 2011) can be readily detected at the *Theileria* schizont surface membrane. Driven by parasite actin polymerization, they extend toward the host cell nucleus or cell periphery (Kuehni-Boghenbor et al., 2012). It is possible that these protrusions could also function to interact with host cell components. The autophagy protein LC3 was detected within and surrounding *Theileria* schizonts in transformed cells, raising the possibility that host and/or *Theileria* autophagy might play a role in parasite survival, although the significance of this observation has not been explored (Latré De Laté et al., 2017). In *Plasmodium*-infected hepatocytes, functional host cell autophagy is required for *Plasmodium* growth and differentiation, perhaps as a source of nutrients for the developing parasite (Thieleke-Matos et al., 2016). On the other hand, recognition of the PVM by autophagic machinery leads to the elimination of the parasite. Live imaging revealed that in the case of successful infection, host autophagy proteins are progressively shed from the *Plasmodium* PVM and accumulate in the tubovesicular network of the PVM (TVN), thus preventing autophagic destruction of the developing parasite (Prado et al., 2015; Agop-Nersesian et al., 2017). How, or if, *Theileria* parasites interact with the host autophagy pathway remains to be seen.

## How Are *Theileria* Proteins Exported Across the Parasite Plasma Membrane?

**FIGURE 3:**
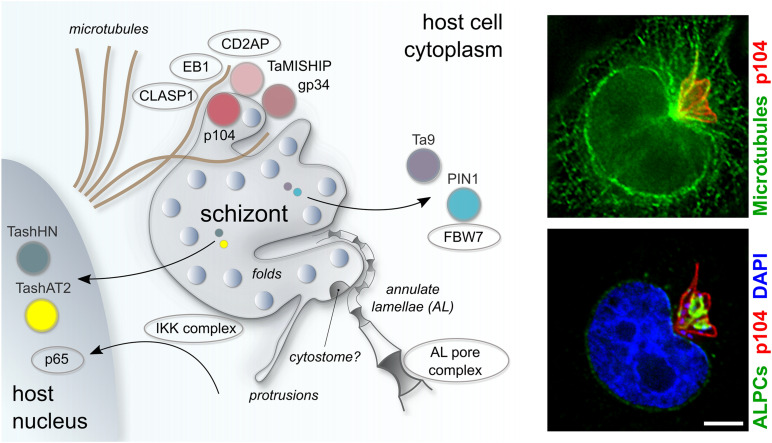
Schematic representation of key host-parasite interactions in *Theileria annulata* infected leukocytes. *Theileria* schizonts export proteins into the host nucleus (TashAT, TashHN) or cytoplasm (PIN1, Ta9), where they have the potential to interact with host proteins (FBW7) to modify the host phenotype. *Theileria* interacts closely with host MTs, mediated in part by a protein complex on the schizont surface comprising bovine CLASP1, CD2AP, EB1 and the schizont proteins p104 and MISHIP. The IKK complex is recruited to the parasite surface, enabling the nuclear localization of NF-kB subunits (p65) to the host nucleus. Porous annulate lamellae that align closely to the schizont membrane are depicted, as are structural elements of the parasite plasma membrane such as dynamic protrusions and a potential cytostome. Fluorescent images show host MTs, labeled with anti-CLASP2 antibodies (green, top panel) and host ALPCs, labeled with anti-RanGAP1 (green, bottom panel) in relation to the schizont membrane, which is labeled with anti-p104 (red). The scale bar is 5 μm.

Although several *Theileria* proteins are known to localize to the host cell cytosol or nucleus in infected cells, the mechanism by which *Theileria* exports proteins into its host cell are not known. Lacking a PV, it is not surprising that *T. annulata* and *T. parva* do not share many orthologs with *T. gondii* and *Plasmodium* spp. protein complexes situated in the PVM and that are involved in the export of effector proteins from the tachyzoite (MYR1 complex; Franco et al., 2016; Marino et al., 2018; Cygan et al., 2020) and red blood cell stage (PTEX complex; de Koning-Ward et al., 2009). The only *Theileria* protein with a homologous sequence to known translocon proteins of other parasites is the putative chaperon protein ClbP (TA07095) which shares sequence identities to HSP101, a member of the PTEX translocon of *Plasmodium* (Beck et al., 2014). TaClbP contains a signal peptide but no transmembrane domain and its localization has not been determined. As HSP101 is expressed in *Plasmodium* liver stage forms (Matthews et al., 2013), its involvement in protein export into the hepatocyte appears likely, although it has not yet been proven. In *Plasmodium* trophozoites, areas of close contact between the PPM and the PVM can be observed, and domains of protein export can be observed on the PVM in these regions (Garten et al., 2020). It is not known whether the PPM of the *Theileria* schizont is similarly structured in patches with distinct functional regions, but the visualization of evenly distributed “knobs” on the PPM by high resolution scanning electron microscopy (SEM) could indicate the accumulation of specialized protein clusters on the parasite surface (Kuehni-Boghenbor et al., 2012). Intriguingly, *Theileria* proteins can form rather large complexes in punctate foci on the membrane surface with multiple host and parasite proteins involved (Figure 1; Xue et al., 2010; Huber et al., 2017, 2018). The most well-studied protein complex present at the schizont surface is the CLASP1/CD2AP/EB1-complex which interacts with the MT system of the host and involves a total of at least seven host and four parasite proteins, including p104 (Figure 3; Huber et al., 2018). The EB1 binding *T. annulata* protein p104 is equipped with a predicted c-terminal GPI-anchor signal sequence and resides on the entire parasite membrane and interacts (directly or indirectly) with TaMISHIP and TA03615, neither of which contain a transmembrane domain but are both found to interact with host proteins on the surface. Several other parasite proteins such as gp34, TaSP and TaSE (TA20205) have been described to also reside on or within the PPM. It is tempting to speculate that parasite proteins incorporated in larger protein complexes are involved in protein, nutrient or small molecule trafficking between the parasite and the host cell cytoplasm. Given the absence of a transport machinery orthologous to those defined in *Plasmodium* or *Toxoplasma*, we consider two attractive possibilities for the export of effector proteins. First, it is possible that no export machinery exists but proteins are exported either passively or via exosome vesicles outside of the schizont confinement into the bovine cell. A role for schizont-derived vesicles being exported into the host cell compartment has not yet been explored. A second possibility is that a *Theileria*-specific translocon apparatus exists, although it has not been identified.

Another question that arises is whether families of effector proteins are actively stored in distinct secretory organelles or if they are continuously synthesized directly before export. In tachyzoites of *T. gondii*, micronemes, rhoptries and dense granules comprise different families of effector proteins. The distinct proteome of these organelles has recently been identified (Barylyuk et al., 2020), though only a limited fraction has been fully characterized. Many *Toxoplasma* and *Plasmodium* proteins targeted for export contain a Texel or HT/Pexel recognition motif, respectively, and these are cleaved by an aspartyl protease (ASP) prior to export (Hiller et al., 2004; Marti et al., 2004; Coffey et al., 2015). A PEXEL-like motif (PLM) has also been identified in *Babesia*, and experiments with recombinant reporters consisting of N-terminal portions of *Babesia* secreted proteins and a fluorescent tag revealed the accumulation of proteins destined for secretion in large vesicular organelles termed “spherical bodies” prior to export into the erythrocyte (Pellé et al., 2015). Although a Pexel or Texel-like motif has not been identified in the *Theileria* genome, it is possible that *Theileria* proteins are processed and cleaved by an ASP prior to storage in distinct organelles and export. *T. annulata* shares an orthologous ASP (TA17685; in *T. parva* TP03_0676) with plasmepsin V (PMV) of *Plasmodium* spp. (Dogga et al., 2017), and it will be interesting to identify the protein targets of this peptidase. *Theileria* sporozoites only harbor rhoptries and microspheres. Though present in some tick stages of *Theileria* (Mehlhorn et al., 1975), apical micronemes are absent from invasive zoites, consistent with the lack of an actin-myosin-based motor complex for active invasion and gliding motility. So far the only described rhoptry protein is p104, first identified as a strongly antigenic protein of 104 kDa in *T. parva*, and detected by immunogold labeling in the rhoptries of sporozoites (Iams et al., 1990b). While *T. annulata* p104 is localized on the PPM of schizonts, immunofluorescence analysis suggests that p104 is, as in *T. parva*, localized in the rhoptries of sporozoites (Huber et al., 2018). Immunogold labeling of *T. parva* sporozoites identified p150 and PIM (named TaSP in *T. annulata*) to be stored in microspheres, which are secreted after invasion (Skilton et al., 1998; Toye et al., 2014). In schizonts, TaSP is expressed on the PPM surface and seems to contribute to the interaction of the schizont with MTs (Seitzer et al., 2010). Intracellular organelles, and in particular storage or secretory vesicles, have not been well characterized in *Theileria* schizonts, although a recent analysis of two HSP90 variants allowed the visualization of the endoplasmic reticulum (TA06470) and the apicoplast (TA10720) of the schizont for the first time (Kinnaird et al., 2017). TEM studies do indicate the presence of electron dense structures within the schizont that resemble rhoptries or dense granules (Figure 2; Shaw and Tilney, 1995). Whether exported protein storage units exist in *Theileria* schizonts, what their contents are, and to what extent the secretion of *Theileria* effector proteins drives cellular transformation, remains to be unraveled.

## The Schizont Surface Is a Signal Transduction Platform

One key signaling pathway that *Theileria* subverts is the NF-κB pathway, and this is achieved by physically recruiting signaling molecules to the schizont surface (Heussler et al., 2002). In *Theileria*-transformed cells, NF-κB is constitutively activated, conferring resistance to apoptosis upon the infected cell. In non-infected cells, NF-κB is retained in the cytoplasm by binding to its cellular inhibitor IκB (inhibitor of κB). The turnover of IkB is regulated by the activity of the IκB kinase complex, IKK (Ghosh and Karin, 2002). In *Theileria* infected cells, IKK complexes, termed signalosomes, are recruited to the schizont surface where they are continually phosphorylated and activated. This leads to the phosphorylation and subsequent degradation of IκB, thus freeing NF-κB subunits for translocation into the nucleus (Heussler et al., 2002). Exactly how IKK is recruited to the parasite surface remains unclear. There is some evidence that the architecture of the actin cytoskeleton in infected cells plays a role in NF-κB activation, and Schmuckli-Maurer and colleagues noticed that in some *T. annulata* infected clones, high NF-κB activity tended to correlate with a looser actin cytoskeleton (Schmuckli-Maurer et al., 2010). In line with this, disruption of the actin cytoskeleton with cytochalasin D or jasplakinolide led to an increase in NF-κB activity. A *T. parva* schizont surface protein termed TpSCOP, absent in the non-transforming *T. orientalis*, was postulated to contribute to NF-κB activation in infected T-cells (Hayashida et al., 2010). TpSCOP binds to host cell F actin via its N-terminal region and partially colocalizes with actin filaments at the schizont surface. Interestingly, over-expression of TpSCOP in mouse T-cells conferred a marked resistance to Fas-mediated apoptosis and led to an increase in phosphorylation of IκB as well as upregulation of NF-κB activity and elevated expression of the anti-apoptotic protein A1/Bfl-1. To date, TpSCOP is the only *Theileria*-encoded protein that has been linked to *Theileria*-dependent transformation via stimulation of NF-κB activity. Infection with *T. gondii* type II strains also leads to an increase in host NF-κB signaling, and recent work showed that activation of the NF-κB pathway is achieved via the interaction between the effector protein GRA15, which is secreted into the PV and localized to the PVM, and TNF receptor-associated factors (TRAFs), adaptor proteins that function upstream of the NF-(κB transcription factor (Sangaré et al., 2019). Simultaneously, *T. gondii* antagonizes the NF-κB signaling pathway by the secreted dense granule protein HCE1/TEEGR (Braun et al., 2019).

Constitutive c-Jun N-terminal kinase (JNK) activity, phosphorylation of c-Jun and activation of the transcription factors ATF-2 and AP-1 has been reported for *T. annulata* and *T. parva* infected lymphocytes, and inhibition of JNK activity or overexpression of JNK mutants result in apoptosis of the infected cell (Baylis et al., 1995; Chaussepied et al., 1998; Adamson et al., 2000; Lizundia et al., 2006). The two major isoforms of JNK, JNK1 and JNK2, exert opposing effects on cell survival. While JNK1 activity increases the stability of c-Jun, JNK2 activity promotes the degradation of c-Jun. Loss of *jnk2* in mouse cells leads to an increase in cell proliferation, while loss of *jnk1* leads to decreased proliferation (Bode and Dong, 2007; Latré De Laté et al., 2019). In *T. annulata* infected macrophages, bovine JNK1 was detected in both the nucleus and cytoplasm of the host, while JNK2 is localized mainly in the cytoplasm and accumulates at the membrane of the schizont (Latré De Laté et al., 2019). *T. annulata* p104 contains three putative JNK-binding motifs and co-precipitates with JNK following immunoprecipitation with anti-pan JNK antibodies. The interaction between p104 and bovine JNK was ablated following treatment with cell-penetrating peptides containing the wild type p104 JNK-binding motif. Importantly, an S > A mutant peptide failed to block the interaction between p104 and JNK, suggesting that the interaction between p104 and JNK2 is promoted by phosphorylation of the S806 and/or S808 residues. Blocking the p104-JNK2 interaction with a penetrating peptide was accompanied by a reduction in JNK1 expression in the nucleus and a reduction in nuclear c-Jun phosphorylation. AP-1 is a transcription factor composed of proteins belonging to the c-Fos, c-Jun, ATF and JDP families. In *Theileria*-infected macrophages, constitutive AP-1 activity drives the transcription of *mmp9*, leading to an increase in matrix metallopeptidase 9 (MMP9) activity. This in turn promotes invasion and dissemination, as measured by matrigel traversal (Adamson et al., 2000; Cock-Rada et al., 2012; Echebli et al., 2014). Following disruption of the p104-JNK2 interaction with the wild type JNK-binding motif peptide, MMP9 activity, as revealed by gelatin gel assay, was reduced and matrigel traversal was significantly decreased. Treatment with the (S > A) mutant peptide as well as irrelevant peptides served as an important control and exerted no effect on matrigel traversal. These data point to a role for p104 in sequestering JNK2 at the parasite surface which might contribute to promoting the survival of the transformed cell by stabilizing c-Jun in the nucleus (Latré De Laté et al., 2019). Secretion of a parasite prolyl isomerase, TaPIN1 (TA18945), has also been implicated in stabilizing c-Jun levels and thus promoting *Theileria*-induced transformation (Marsolier et al., 2015), and will be discussed in the next section.

P53 is a nuclear transcription factor that functions to drive apoptosis in response to stress or DNA damage, thus protecting cells from accumulating genomic aberrations that can lead to cancer. In many tumors, the tumor suppressor protein p53 is mutated, leading to a loss of function (Ozaki and Nakagawara, 2011). In *Theileria*-transformed cells p53 activity has also been reported to be low, and two different mechanisms by which the parasite achieves this have been proposed (Haller et al., 2010). Haller and colleagues detected p53 decorating the surface of the schizont in *T. annulata* infected macrophages. They proposed that the sequestration of p53 at the schizont surface prevents its translocation to the nucleus, and in line with this observation, p53-transcribed apoptotic genes were found to be regulated in a parasite-dependent manner. The expression of the anti-apoptotic protein Bcl-2 increases upon buparvaquone treatment, while the expression of pro-apoptotic proteins Apaf-1 and Bax decreases upon killing of the parasite with buparvaquone treatment (Haller et al., 2010). In another study, no schizont surface localization of p53 could be detected in *T. parva* infected T cells (Hayashida et al., 2013). Instead, those authors found that MDM2, a well-characterized oncoprotein, is expressed at high levels in *T. parva* infected lymphocytes. MDM2 protein is a negative regulator of p53 activity that controls p53 expression at both the transcriptional and translational level as well as by regulating proteasomal degradation of p53. *T. parva* infected lymphocytes are sensitive to MDM2 inhibitors such as TIBC and nutlin-3, whereas non-infected ConA-stimulated lymphocytes were insensitive. In *T. parva* infected lymphocytes, protein levels of p53 are low and do not increase in response to DNA damaging agents such as cisplatin, even as transcript levels of p53 increase. Treatment of infected cells with the MDM2 inhibitor TIBC led to nuclear accumulation of p53, and restoration of p53-targeted pro-apoptotic molecules such as Bax. Together these data led to the conclusion that highly expressed MDM2 in *T. parva* infected cells contributes to suppression of p53 protein expression by targeting p53 for proteasomal degradation. Variable expression levels of MDM2 observed in different *Theileria*-infected clones (Hayashida et al., 2013) might lead to varied p53 expression and could explain the difficulty in detecting p53 at the schizont surface in some *T. parva* and *T. annulata* infected cell lines (Hayashida et al., 2013). While it is clear that several changes observed in Theileria-infected cells, including alterations to p53 and NF-κB pathways, resemble those seen with cellular transformation in cancer, it has not known how soon following transformation these alterations occur. A transcriptomic or proteomic analysis of freshly infected and transformed cell lines will help to unravel whether these alterations drive *Theileria*-dependent transformation, or are in fact acquired subsequently to parasite-induced transformation, as occurs with secondary mutations in many cancers (Brown et al., 2019).

## Secretion of *Theileria*-Encoded Effector Proteins Into Host Cell Compartments

Several *Theileria* effector proteins, defined as proteins of transformative *Theileria* species which are translocated into the host cell or integrated into the schizont membrane and interact with host proteins, have been described in recent years (reviewed in Tajeri and Langsley, 2021). Some proteins have been shown to translocate to the host cell nucleus, and key examples are three Tash family proteins TashAT2 (TA20095), TashHN (TA20090) and SuAT1 (TA03135) (Swan et al., 1999; Swan et al., 2001; Swan et al., 2003; Shiels et al., 2004). Others reside in the host cell cytoplasm, as it is the case for Ta9 (TA15705) and TaPIN1 (TA18945), both of which have been implicated in the proliferative phenotype of the host cell (Marsolier et al., 2015, 2019; Unlu et al., 2018). *T. annulata* and *T. parva* genomes contain multiple expanded protein families, which are absent in non-transformative species such as *T. orientalis*. The Tash and Ta9 protein families are both examples of such gene families. The Tash family encompasses 17 genes in *T. annulata* and 20 genes in *T. parva* (Pain et al., 2005). All Tash family proteins contain an N-terminal signal sequence and some contain an AT hook DNA-binding domain (Swan et al., 1999; Swan et al., 2001). Eight direct orthologous gene pairs can be found located at either end of the gene cluster of the Tash genes between *T. annulata* and *T. parva*. However, in the middle of the cluster a significant species-specific diversification is visible (Pain et al., 2005; Shiels et al., 2006; Weir et al., 2009). Following overexpression of *TashAT2* in non-infected bovine cells (BoMac), changes in morphology became apparent although no difference in proliferation was observed. In *TashAT2*-transfected BoMac cells transcripts for the mitochondrial genes cytochrome oxidase and NADH dehydrogenase were upregulated compared to cells expressing a control plasmid, while expression of the ubiquitin-like protease UBP43 and its ubiquitin-like substrate, bISG15, were significantly reduced in TashAT2-expressing cells (Oura et al., 2006). The ISGylation system, comprising of UBP43 and ISG15 together with UBE1L and Ubc8, plays a role in cellular defense against viral and bacterial infection (Ritchie et al., 2004; Kim et al., 2005), and is strongly induced by type 1 interferons (IFNs) and lipopolysaccharide (LPS). Although UBP43 and ISG15 are expressed more strongly in *Theileria*-infected cells compared to their uninfected counterparts, infected cells were found to be refractory to LPS- and IFN-α stimulated induction of very high levels of ISG15 and UBP43. This led to a tentative suggestion that the parasite dependent repression of elevated ISG15 levels might allow *Theileria* to escape from a protective immune response (Oura et al., 2006). Overexpression of SuAT1 in BoMac cells induced a change in gene expression for cytoskeletal polypeptides and caused a similar change in morphology to that seen upon overexpression of TashAT2, with cells becoming larger and more spread out (Shiels et al., 2004).

The Ta9 family protein cluster is located on chromosome 2 with five members in *T. annulata* and six members in *T. parva*. In the non-transforming *Theileria* species *T. orientalis*, only one protein with weak homology to Ta9 has been identified in the syntenic region on chromosome 2 (Hayashida et al., 2012). Ta9 has been shown to localize to the host cell cytoplasm by immunofluorescence analysis in *T. annulata* (Unlu et al., 2018) and is one of the main CD8 (T cell antigens in both T. annulata and T. parva infection (MacHugh et al., 2011). Expression of Ta9 increases during schizont development and is reduced again at the onset of merogony (Hayashida et al., 2012), suggesting a role in the manipulation of the host cell during the schizont stage. Luciferase reporter assays in Ta9-expressing HEK293T cells indicate that Ta9 expression might activate AP-1 driven transcription (Unlu et al., 2018). However, this has not been shown in bovine cell lines or parasitized cells and, although the relevant polypeptide sequence has been narrowed down to the C-terminal region of the Ta9 protein, the mechanism by which AP-1 activation occurs is still unknown. A second protein which has been detected in the host cell cytoplasm is the *T. annulata* peptidyl-prolyl isomerase 1 (TaPin1). TaPin1 was identified by a comparative genomics approach and subsequent characterization showed that it interacts with the host ubiquitin ligase FBW7 and might influence the host c-Jun signaling pathway (Marsolier et al., 2015). C-Jun signaling is involved in oncogenic transformation and has been previously shown to be modulated by *Theileria* schizonts (Lizundia et al., 2006). Further analysis of TaPin1 revealed that the *T. annulata* protein also interacts with the host pyruvate kinase isoform M2 (PKM2), and elevated levels of PKM2 were shown in parasitized lymphocytes compared to non-infected cells (Marsolier et al., 2019). As a cofactor of the transcription factor hypoxia-inducible factor 1 alpha (HIF1α), PKM2 plays an important role in the transcriptional control of glycolytic enzymes in cancer cells (Wang et al., 2014). These enzymes are responsible for the Warburg effect, a metabolic alteration toward aerobic glycolysis which is also observed in *Theileria*-infected lymphocytes (Medjkane and Weitzman, 2013; Medjkane et al., 2014; Metheni et al., 2015). Interestingly, transcriptional activity of HIF1α and expression of HIF1α target genes is reduced in parasitized cells upon treatment with the theilericidal drug buparvaquone. This loss of activity was ascribed to the prolyl isomerase activity of TaPin1 and can be partially rescued by expressing exogenous TaPin1 in the parasitized cells. It is likely that only a small fraction of the potential arsenal of *Theileria*-encoded secreted effector proteins have been identified to date. Whether *Theileria* encodes a single “master regulator” protein that drives host transformation, or whether multiple secreted schizont-encoded proteins work together to modulate the host phenotype, remains to be seen.

## Concluding Remarks

*Theileria* schizonts have the unique ability to induce the transformation of their host cell, leading to a significant remodeling of the host phenotype and the clonal expansion of parasitized leukocytes. A striking aspect of *Theileria* biology is the close interaction the schizont maintains with host MTs during its intracellular life. The mimicking of particular protein binding motifs, such as the EB1-binding SxIP motif in the schizont surface protein p104, demonstrates the elegant mechanism used by the parasite to ensure its distribution to daughter cells during host mitosis and cytokinesis. A remarkable degree of co-dependency exists between the schizont and the transformed leukocyte. Gene expression studies have revealed that the parasite irreversibly rewires gene expression patterns in the host (Kinnaird et al., 2013), and killing the parasite with the potent and specific anti-*theilerial* drug buparvaquone leads to a halt in proliferation and the onset of apoptotic cell death. The close relationship between the intracellular schizont and the leukocyte brings with it a number of challenges and raises some intriguing questions on how *Theileria* induces such fundamental changes.

First, how does the schizont drive cellular transformation, and what effector proteins are involved? To date, only few *Theileria*-encoded secreted proteins that can interact with the host have been characterized, key examples being Tash family proteins (Swan et al., 1999, 2001, 2003; Shiels et al., 2004), a parasite-encoded prolyl-isomerase Pin1 (Marsolier et al., 2015, 2019), and Ta9, a secreted protein that is unique to transforming *Theileria* species (Unlu et al., 2018). Unfortunately, with the lack of an efficient method to genetically manipulate *Theileria*, functional studies into the mode of action of potential *Theileria* effectors are limited. A great hope for the future is that with the availability of efficient CRISPR-Cas9 gene editing tools, a robust transfection method will be established and will open the field for mechanistic investigations into *Theileria*-host interactions. *Theileria* modulates the host phenotype not only by secreting proteins to the host cytoplasm or nucleus, but also by recruiting host signaling molecules to its surface. Some interesting examples are the recruitment and activation of IKK signalosomes at the PPM, leading to anti-apoptotic NF-kB signaling (Heussler et al., 2002), or the p104-mediated binding of JNK2 to the parasite surface that might contribute to c-Jun activation (Latré De Laté et al., 2019). However, the details of the mechanisms by which JNK2 and IKK are sequestered at the PPM have not been fully uncovered.

Further, how parasite effector proteins are stored within the schizont and exported into the host remains unknown. Does *Theileria* possess a translocon-like complex on its membrane that facilitates protein export? In this context the presence of knob-like structures on the schizont PPM, as well as the identification of the CLASP1-CD2AP-EB1-p104-TaMISHIP protein complex in punctate foci on the membrane is interesting, although further investigation into protein complexes at the PPM is required. Another question that remains unexplored is if and how *Theileria* schizonts scavenge nutrients from the host. In particular, the frequent observation of a cytostome-like structure in close proximity to AL hints at the mechanism for uptake of lipids and other molecules. Little is known about the potential recruitment of host organelles to the schizont surface, although the close alignment of porous AL and associated nuclear transport machinery with the schizont membrane is intriguing. Ongoing studies investigating the host-pathogen interactions in *Theileria*-transformed leukocytes will hopefully yield fascinating insights into the survival strategies of this remarkable pathogen.

## Author Contributions

KW and PO developed the ideas for the manuscript. KW, CP, FB, and PO all contributed to writing and editing. All authors contributed to the article and approved the submitted version.

## Conflict of Interest

The authors declare that the research was conducted in the absence of any commercial or financial relationships that could be construed as a potential conflict of interest. The handling editor declared a shared affiliation with the authors at the time of review.

## References

[B1] Adamson R. Logan M. Kinnaird J. Langsley G. Hall R. (2000). Loss of matrix metalloproteinase 9 Activity in *Theileria annulata*-attenuated cells is at the transcriptional level and is associated with differentially expressed AP-1 species. *Mol. Biochem. Parasitol.* 106 51–61. 10.1016/s0166-6851(99)00213-3 10743610

[B2] Agop-Nersesian C. De Niz M. Niklaus L. Prado M. Eickel N. Heussler V. T. (2017). Shedding of host autophagic proteins from the parasitophorous vacuolar membrane of *Plasmodium berghei*. *Sci. Rep.* 7:2191. 10.1038/s41598-017-02156-7 28526861 PMC5438358

[B3] Aguirre A. A. Longcore T. Barbieri M. Dabritz H. Hill D. Klein P. N. (2019). The one health approach to toxoplasmosis: epidemiology, control, and prevention strategies. *Ecohealth* 16 378–390. 10.1007/s10393-019-01405-7 30945159 PMC6682582

[B4] Al-Bassam J. Kim H. Brouhard G. van Oijen A. Harrison S. C. Chang F. (2010). CLASP promotes microtubule rescue by recruiting tubulin dimers to the microtubule. *Dev. Cell* 19 245–258. 10.1016/j.devcel.2010.07.016 20708587 PMC3156696

[B5] Bargieri D. Y. Andenmatten N. Lagal V. Thiberge S. Wang J. A. Tardieux I. (2013). Apical membrane Antigen 1 mediates apicomplexan parasite attachment but is dispensable for host cell invasion. *Nat. Commun.* 4:2552. 10.1038/ncomms3552 24108241 PMC3826631

[B6] Barylyuk K. Koreny L. Ke H. Butterworth S. Crook O. M. Lassadi I. (2020). A comprehensive subcellular atlas of the *Toxoplasma* proteome via hyperLOPIT provides spatial context for protein functions. *Cell Host Microb.* 28 752–766.e9. 10.1016/j.chom.2020.09.011 PMC7670262 33053376

[B7] Batista M. F. Nájera C. A. Meneghelli I. Bahia D. (2020). The parasitic intracellular lifestyle of trypanosomatids: parasitophorous vacuole development and survival. *Front. Cell Dev. Biol.* 8:396. 10.3389/fcell.2020.00396 32587854 PMC7297907

[B8] Baylis H. A. Megson A. Hall R. (1995). Infection with *Theileria annulata* induces expression of matrix Metalloproteinase 9 and transcription factor AP-1 in bovine leucocytes. *Mol. Biochem. Parasitol.* 69 211–222. 10.1016/0166-6851(94)00216-a 7770085

[B9] Beck J. R. Muralidharan V. Oksman A. Goldberg D. E. (2014). PTEX component HSP101 mediates export of diverse malaria effectors into host erythrocytes. *Nature* 511 592–595. 10.1038/nature13574 25043010 PMC4130291

[B10] Bode A. M. Dong Z. (2007). The functional contrariety of JNK. *Mol. Carcinogen.* 46 591–598. 10.1002/mc.20348 17538955 PMC2832829

[B11] Bohnert M. (2020). Tether Me, Tether Me Not—dynamic organelle contact sites in metabolic rewiring. *Dev. Cell* 54 212–225. 10.1016/j.devcel.2020.06.026 32693056

[B12] Braun L. Brenier-Pinchart M.-P. Hammoudi P.-M. Cannella D. Kieffer-Jaquinod S. Vollaire J. (2019). The *Toxoplasma* effector TEEGR Promotes parasite persistence by modulating NF-K B signalling via EZH2. *Nat. Microbiol.* 4 1208–1220. 10.1038/s41564-019-0431-8 31036909 PMC6591128

[B13] Brown A.-L. Li M. Goncearenco A. Panchenko A. R. (2019). Finding driver mutations in cancer: elucidating the role of background mutational processes. *PLoS Computat. Biol.* 15:e1006981. 10.1371/journal.pcbi.1006981 31034466 PMC6508748

[B14] Chaussepied M. Lallemand D. Moreau M.-F. Adamson R. Hall R. Langsley G. (1998). Upregulation of Jun and Fos family members and permanent JNK activity lead to constitutive AP-1 activation in *Theileria*-transformed leukocytes. *Mol. Biochem. Parasitol.* 94 215–226. 10.1016/s0166-6851(98)00070-x 9747972

[B15] Cheeseman K. Weitzman J. B. (2015). Host-parasite interactions: an intimate epigenetic relationship: epigenetics in apicomplexa infections. *Cell. Microbiol.* 17 1121–1132. 10.1111/cmi.12471 26096716

[B16] Cock-Rada A. M. Medjkane S. Janski N. Yousfi N. Perichon M. Chaussepied M. (2012). SMYD3 promotes cancer invasion by epigenetic upregulation of the Metalloproteinase MMP-9. *Cancer Res.* 72 810–820. 10.1158/0008-5472.can-11-1052 22194464 PMC3299564

[B17] Coffey M. J. Sleebs B. E. Uboldi A. D. Garnham A. Franco M. Marino N. D. (2015). An aspartyl protease defines a novel pathway for export of *Toxoplasma* proteins into the host Cell. *eLife* 4:e10809. 10.7554/eLife.10809 PMC4764566 26576949

[B18] Coppens I. Dunn J. D. Romano J. D. Pypaert M. Zhang H. Boothroyd J. C. (2006). *Toxoplasma gondii* sequesters lysosomes from mammalian hosts in the vacuolar space. *Cell* 125 261–274. 10.1016/j.cell.2006.01.056 16630815

[B19] Coppens I. Romano J. D. (2018). Correction: hostile intruder: *Toxoplasma* holds host organelles captive. *PLoS Pathog.* 14:e1007018. 10.1371/journal.ppat.1007018 29596535 PMC5875880

[B20] Cygan A. M. Theisen T. C. Mendoza A. G. Marino N. D. Panas M. W. Boothroyd J. C. (2020). Coimmunoprecipitation with MYR1 identifies three additional proteins within the *Toxoplasma gondii* parasitophorous vacuole required for translocation of dense granule effectors into host cells. *mSphere* 5:e00858-19. 10.1128/mSphere.00858-19 PMC7031616 32075880

[B21] D’Angelo M. A. Hetzer M. W. (2008). Structure, dynamics and function of nuclear pore complexes. *Trends Cell Biol.* 18 456–466. 10.1016/j.tcb.2008.07.009 18786826 PMC2697926

[B22] de Koning-Ward T. F. Gilson P. R. Boddey J. A. Rug M. Smith B. J. Papenfuss A. T. (2009). A newly discovered protein export machine in malaria parasites. *Nature* 459 945–949. 10.1038/nature08104 19536257 PMC2725363

[B23] Dikic I. (2002). CIN85/CMS family of adaptor molecules. *FEBS Lett.* 529 110–115. 10.1016/S0014-5793(02)03188-5 12354621

[B24] Dobbelaere D. A. Shapiro S. Z. Webster P. (1985). Identification of a surface antigen on T*heileria parva* sporozoites by monoclonal antibody. *Proc. Natl. Acad. Sci. U.S.A.* 82 1771–1775. 10.1073/pnas.82.6.1771 3920654 PMC397354

[B25] Dobbelaere D. A. E. Rottenberg S. (2003). Theileria-induced leukocyte transformation. *Curr. Opin. Microbiol.* 6 377–382. 10.1016/s1369-5274(03)00085-7 12941408

[B26] Dogga S. K. Mukherjee B. Jacot D. Kockmann T. Molino L. Hammoudi P.-M. (2017). A druggable secretory protein maturase of *Toxoplasma* essential for invasion and egress. *eLife* 6:e27480. 10.7554/eLife.27480 PMC5595437 28898199

[B27] Dundas K. Shears M. J. Sinnis P. Wright G. J. (2019). Important extracellular interactions between *Plasmodium* sporozoites and host cells required for infection. *Trends Parasitol.* 35 129–139. 10.1016/j.pt.2018.11.008 30583849 PMC6375296

[B28] Echebli N. Chaussepied M. M. M. Vayssettes C. Santo J. P. D. Darghouth M. A. Langsley G. (2014). Engineering attenuated virulence of a *Theileria annulata*-infected macrophage. *PLoS Negl. Trop. Dis.* 8:e3183. 10.1371/journal.pntd.0003183 25375322 PMC4222746

[B29] Fawcett D. Musoke A. Voigt W. (1984). Interaction of sporozoites of *Theileria parva* with bovine lymphocytes in Vitro. 1. Early events after invasion. *Tissue Cell* 16 873–884. 10.1016/0040-8166(84)90068-5 6442474

[B30] Fawcett D. W. Doxsey S. Stagg D. A. Young A. S. (1982). The entry of sporozoites of *Theileria parva*into bovine lymphocytes in vitro. Electron microscopic observations. *Eur. J. Cell Biol.* 27 10–21. 6806100

[B31] Flynn D. C. (2001). Adaptor proteins. *Oncogene* 20 6270–6272. 11607828 10.1038/sj.onc.1204769

[B32] Franco M. Panas M. W. Marino N. D. Lee M.-C. W. Buchholz K. R. Kelly F. D. (2016). A novel secreted protein, MYR1, Is Central to *Toxoplasma’s* manipulation of host cells. *mBio* 7:e02231-15. 10.1128/mBio.02231-15 PMC4742717 26838724

[B33] Frénal K. Dubremetz J.-F. Lebrun M. Soldati-Favre D. (2017). Gliding motility powers invasion and egress in apicomplexa. *Nat. Rev. Microbiol.* 15 645–660. 10.1038/nrmicro.2017.86 28867819

[B34] Frischknecht F. Matuschewski K. (2017). *Plasmodium* sporozoite biology. *Cold Spring Har. Perspect. Med.* 7:a025478. 10.1101/cshperspect.a025478 28108531 PMC5411682

[B35] Garten M. Beck J. R. Roth R. Tenkova-Heuser T. Heuser J. Istvan E. S. (2020). Contacting domains segregate a lipid transporter from a solute transporter in the malarial host-parasite interface. *Nat. Commun.* 11:3825. 10.1038/s41467-020-17506-9 PMC7393353 32732874

[B36] Ghosh S. Karin M. (2002). Missing pieces in the NF-K B Puzzle. *Cell* 109 S81–S96. 11983155 10.1016/s0092-8674(02)00703-1

[B37] Glass E. J. Crutchley S. Jensen K. (2012). Living with the enemy or uninvited guests: functional genomics approaches to investigating host resistance or tolerance traits to a protozoan parasite, *Theileria annulata*, in cattle. *Vet. Immunol. Immunopathol.* 148 178–189. 10.1016/j.vetimm.2012.03.006 22482839 PMC7112524

[B38] Gouveia S. M. Akhmanova A. (2010). Cell and molecular biology of microtubule plus end tracking proteins. *Int. Rev. Cell Mol. Biol.* 285 1–74. 10.1016/B978-0-12-381047-2.00001-3 21035097

[B39] Guérin A. Corrales R. M. Parker M. L. Lamarque M. H. Jacot D. Hajj H. E. (2017). Efficient invasion by *Toxoplasma* depends on the subversion of host protein networks. *Nat. Microbiol.* 2 1358–1366. 10.1038/s41564-017-0018-1 28848228

[B40] Hall R. Boulter N. R. Brown C. G. D. Wilkie G. Kirvar E. Nene V. (2000). Reciprocal cross-protection induced by sporozoite antigens SPAG-1 from theileria annulata and P67 from *Theileria parva*. *Parasite Immunol.* 22 223–230. 10.1046/j.1365-3024.2000.00302.x 10792761

[B41] Haller D. Mackiewicz M. Gerber S. Beyer D. Kullmann B. Schneider I. (2010). Cytoplasmic sequestration of P53 promotes survival in leukocytes transformed by *Theileria*. *Oncogene* 29 3079–3086. 10.1038/onc.2010.61 20208567

[B42] Hayashida K. Hara Y. Abe T. Yamasaki C. Toyoda A. Kosuge T. (2012). Comparative genome analysis of three eukaryotic parasites with differing abilities to transform leukocytes reveals key mediators of *Theileria*-induced leukocyte transformation. *mBio* 3:e0204-12. 10.1128/mBio.00204-12 PMC3445966 22951932

[B43] Hayashida K. Hattori M. Nakao R. Tanaka Y. Kim J.-Y. Inoue N. (2010). A schizont-derived protein, TpSCOP, is involved in the activation of NF-Kappa B in *Theileria parva*-infected lymphocytes. *Mol. Biochem. Parasitol.* 174 8–17. 10.1016/j.molbiopara.2010.06.005 20540970

[B44] Hayashida K. Kajino K. Hattori M. Wallace M. Morrison I. Greene M. I. (2013). MDM2 regulates a novel form of incomplete neoplastic transformation of *Theileria parva* infected lymphocytes. *Exper. Mol. Pathol.* 94 228–238. 10.1016/j.yexmp.2012.08.008 22981919

[B45] Hehl A. B. Lekutis C. Grigg M. E. Bradley P. J. Dubremetz J.-F. Ortega-Barria E. (2000). toxoplasma gondii homologue of *Plasmodium* apical membrane antigen 1 is involved in invasion of host cells. *Infect. Immun.* 68 7078–7086. 10.1128/IAI.68.12.7078-7086.2000 11083833 PMC97818

[B46] Heussler V. T. Rottenberg S. Schwab R. Küenzi P. Fernandez P. C. McKellar S. (2002). Hijacking of host Cell IKK signalosomes by the transforming parasite *Theileria*. *Science* 298 1033–1036. 10.1126/science.1075462 12411708

[B47] Hiller N. L. Bhattacharjee S. van Ooij C. Liolios K. Harrison T. Lopez-Estraño C. (2004). A Host-targeting signal in virulence proteins reveals a secretome in malarial infection. *Science* 306 1934–1937. 10.1126/science.1102737 15591203

[B48] Huber S. Bär A. Epp S. Schmuckli-Maurer J. Eberhard N. Humbel B. M. (2020). Recruitment of host nuclear pore components to the vicinity of *Theileria* schizonts. *mSphere* 5:e0709-19. 10.1128/mSphere.00709-19 32024710 PMC7002307

[B49] Huber S. Karagenc T. Ritler D. Rottenberg S. Woods K. (2018). Identification and characterisation of a *Theileria annulata* proline-rich microtubule and SH3 domain-interacting protein (TaMISHIP) that forms a complex with CLASP1, EB1, and CD2AP at the schizont surface. *Cell. Microbiol.* 20:e12838. 10.1111/cmi.12838 29520916 PMC6033098

[B50] Huber S. Theiler R. de Quervain D. Wiens O. Karangenc T. Heussler V. (2017). The microtubule-stabilizing protein CLASP1 associates with the T*heileria annulata* schizont surface via its kinetochore-binding domain. *mSphere* 2:e00215-17. 10.1128/mSphere.00215-17 PMC5566832 28861517

[B51] Hulliger L. Wilde J. K. H. Brown C. G. D. Turner L. (1964). Mode of multiplication of *Theileria* in cultures of bovine lymphocytic cells. *Nature* 203:728. 10.1038/203728a0 14207267

[B52] Huynh M.-H. Carruthers V. B. (2006). *Toxoplasma* MIC2 is a major determinant of invasion and virulence. *PLoS Pathog.* 2:e84. 10.1371/journal.ppat.0020084 16933991 PMC1550269

[B53] Iams K. P. Hall R. Webster P. Musoke A. J. (1990a). Identification of lambda Gt11 clones encoding the major antigenic determinants expressed by *Theileria parva* sporozoites. *Infect. Immun.* 58 1828–1834. 10.1128/iai.58.6.1828-1834.1990 1692810 PMC258731

[B54] Iams K. P. Young J. R. Nene V. Desai J. Webster P. Ole-MoiYoi O. K. (1990b). Characterisation of the gene encoding a 104-Kilodalton micronemerhoptry protein of *Theileria parva*. *Mol. Biochem. Parasitol.* 39 47–60. 10.1016/0166-6851(90)90007-9 1689460

[B55] Irvin A. D. Ocgama J. G. R. Spooner P. R. (1982). Cycle of bovine lymphoblastoid cells parasitized by *Theileria parva*. *Res. Vet. Sci.* 33 298–304. 10.1016/s0034-5288(18)32305-1 6818647

[B56] Jain A. Holthuis J. C. M. (2017). Membrane contact sites, ancient and central hubs of cellular lipid logistics. *Biochim. Biophys. Acta* 1864 1450–1458. 10.1016/j.bbamcr.2017.05.017 28554771

[B57] Jalovecka M. Hajdusek O. Sojka D. Kopacek P. Malandrin L. (2018). The complexity of piroplasms life cycles. *Front. Cell. Infect. Microbiol.* 8:248. 10.3389/fcimb.2018.00248 30083518 PMC6065256

[B58] Jura W. G. Brown C. G. Rowland A. C. (1983). Ultrastructural characteristics of in vitro parasite-lymphocyte behaviour in invasions with *Theileria annulata* and *Theileria Parva*. *Vet. Parasitol.* 12 115–134. 10.1016/0304-4017(83)90001-8 6412424

[B59] Kessel R. G. (1992). Annulate lamellae: a last frontier in cellular organelles. *Intern. Rev. Cytol.* 133 43–120. 10.1016/s0074-7696(08)61858-6 1374369

[B60] Kim K. I. Malakhova O. A. Hoebe K. Yan M. Beutler B. Zhang D.-E. (2005). Enhanced antibacterial potential in UBP43-Deficient mice against *Salmonella Typhimurium* infection by up-regulating Type I IFN signaling. *J. Immunol.* 175 847–854. 10.4049/jimmunol.175.2.847 16002682

[B61] Kinnaird J. H. Singh M. Gillan V. Weir W. Calder E. D. D. Hostettler I. (2017). Characterization of HSP90 isoforms in transformed bovine leukocytes infected with *Theileria annulata*. *Cell. Microbiol.* 19:e12669. 10.1111/cmi.12669 27649068 PMC5333456

[B62] Kinnaird J. H. Weir W. Durrani Z. Pillai S. S. Baird M. Shiels B. R. (2013). A Bovine lymphosarcoma cell line infected with *Theileria annulata* exhibits an irreversible reconfiguration of host cell gene expression. *PLoS One* 8:e66833. 10.1371/journal.pone.0066833 23840536 PMC3694138

[B63] Kuehni-Boghenbor K. Ma M. Lemgruber L. Cyrklaff M. Frischknecht F. Gaschen V. (2012). Actin-mediated plasma membrane plasticity of the intracellular parasite *Theileria annulata*. *Cell. Microbiol.* 14 1867–1879. 10.1111/cmi.12006 22891986

[B64] Latré De Laté P. Haidar M. Ansari H. Tajeri S. Szarka E. Alexa A. (2019). *Theileria* highjacks JNK2 into a complex with the macroschizont GPI (GlycosylPhosphatidylInositol)-anchored surface protein P104. *Cell. Microbiol.* 21:e12973. 10.1111/cmi.12973 30412643

[B65] Latré De Laté P. Pineda M. Harnett M. Harnett W. Besteiro S. Langsley G. (2017). Apicomplexan autophagy and modulation of autophagy in parasite-infected host cells. *Biomed. J.* 40 23–30. 10.1016/j.bj.2017.01.001 28411879 PMC6138587

[B66] Leano J. B. Slep K. C. (2019). Structures of TOG1 and TOG2 from the human microtubule dynamics regulator CLASP1. *PLoS One* 14:e0219823. 10.1371/journal.pone.0219823 31323070 PMC6641166

[B67] Li S.-A. Liu L. Guo X.-L. Zhang Y.-Y. Xiang Y. Wang Q.-Q. (2017). Host pore-forming protein complex neutralizes the acidification of endocytic organelles to counteract intracellular pathogens. *J. Infect. Dis.* 215 1753–1763. 10.1093/infdis/jix183 28419297

[B68] Lizundia R. Chaussepied M. Huerre M. Werling D. Di Santo J. P. Langsley G. (2006). C-Jun NH_2_-Terminal Kinase/c-Jun signaling promotes survival and Metastasis of B Lymphocytes transformed by *Theileria*. *Cancer Res.* 66 6105–6110. 10.1158/0008-5472.can-05-3861 16778183

[B69] MacHugh N. D. Weir W. Burrells A. Lizundia R. Graham S. P. Taracha E. L. (2011). Extensive polymorphism and evidence of immune selection in a highly dominant antigen recognized by bovine CD8 T cells specific for *Theileria annulata*. *Infect. Immun.* 79 2059–2069. 10.1128/iai.01285-10 21300773 PMC3088144

[B70] Mackiewicz M. Seitzer U. Ahmed J. S. Reiling N. (2020). Theileria annulata surface protein (TaSP) is a target of cyclin-dependent Kinase 1 phosphorylation in theileria annulata-infected cells. *Transbound. Emerg. Dis.* 67 40–55. 10.1111/tbed.13458 32174040

[B71] Maiato H. Fairley E. A. L. Rieder C. L. Swedlow J. R. Sunkel C. E. Earnshaw W. C. (2003). Human CLASP1 is an outer kinetochore component that regulates spindle microtubule dynamics. *Cell* 113 891–904. 10.1016/S0092-8674(03)00465-3 12837247

[B72] Marino N. D. Panas M. W. Franco M. Theisen T. C. Naor A. Rastogi S. (2018). Identification of a novel protein complex essential for effector translocation across the parasitophorous vacuole membrane of T*oxoplasma gondii*. *PLoS Pathog.* 14:e1006828. 10.1371/journal.ppat.1006828 29357375 PMC5794187

[B73] Marsolier J. Perichon M. DeBarry J. D. Villoutreix B. O. Chluba J. Lopez T. (2015). *Theileria* parasites secrete a prolyl isomerase to maintain host leukocyte transformation. *Nature* 520 378–382. 10.1038/nature14044 25624101 PMC4401560

[B74] Marsolier J. Perichon M. Weitzman J. B. Medjkane S. (2019). Secreted parasite pin1 isomerase stabilizes host pkm2 to reprogram host cell metabolism. *Commun. Biol.* 2:152. 10.1038/s42003-019-0386-6 PMC6491484 31044177

[B75] Marti M. Good R. T. Rug M. Knuepfer E. Cowman A. F. (2004). Targeting malaria virulence and remodeling proteins to the host erythrocyte. *Science* 306 1930–1933. 10.1126/science.1102452 15591202

[B76] Matthews K. Kalanon M. Chisholm S. A. Sturm A. Goodman C. D. Dixon M. W. A. (2013). The *Plasmodium* translocon of exported proteins (PTEX) component Thioredoxin-2 is important for maintaining normal blood-stage growth. *Mol. Microbiol.* 89 1167–1186. 10.1111/mmi.12334 23869529

[B77] Medjkane S. Perichon M. Marsolier J. Dairou J. Weitzman J. B. (2014). *Theileria* induces oxidative stress and HIF1α activation that are essential for host leukocyte transformation. *Oncogene* 33 1809–1817. 10.1038/onc.2013.134 23665677

[B78] Medjkane S. Weitzman J. B. (2013). A reversible warburg effect is induced by *Theileria* parasites to transform host leukocytes. *Cell Cycle* 12 2167–2168. 10.4161/cc.25540 23803730 PMC3755061

[B79] Mehlhorn H. Weber G. Schein E. Büscher G. (1975). Elektronenmikroskopische untersuchung an entwicklungsstadien von *Theileria annulata* (Dschunkowsky, Luhs, 1904) im Darm und in der Hämolymphe von Hyalomma anatolicum excavatum (Koch, 1844). *Zeitschrift für Parasitenkunde* 48 137–150. 10.1007/bf00389644 814733

[B80] Metheni M. Lombès A. Bouillaud F. Batteux F. Langsley G. (2015). HIF-1α induction, proliferation and glycolysis of *Theileria* -infected leukocytes: HIF-1α induction, proliferation and glycolysis. *Cell. Microbiol.* 17 467–472. 10.1111/cmi.12421 25620534

[B81] Mimori-Kiyosue Y. Grigoriev I. Sasaki H. Matsui C. Akhmanova A. Tsukita S. (2006). Mammalian CLASPs are required for mitotic spindle organization and kinetochore alignment. *Genes Cells* 11 845–857. 10.1111/j.1365-2443.2006.00990.x 16866869

[B82] Morrison W. I. (2015). . The aetiology, pathogenesis and control of theileriosis in domestic animals. *Revue Sci. Tech. Off. Intern. Des. Epizoot.* 34 599–611. 10.20506/rst.34.2.2383 26601460

[B83] Musoke A. (1992). A Recombinant sporozoite surface antigen of *Theileria parva* induces protection in cattle. *Proc. Natl. Acad. Sci. U.S.A.* 5 514–518. 10.1073/pnas.89.2.514 1731322 PMC48269

[B84] ole-MoiYoi O. K. Brown W. C. Iams K. P. Nayar A. Tsukamoto T. Macklin M. D. (1993). Evidence for the induction of casein Kinase II in bovine Lymphocytes transformed by the intracellular protozoan parasite *Theileria parva*. *EMBO J.* 12 1621–1631. 10.1002/j.1460-2075.1993.tb05807.x 8467809 PMC413376

[B85] Oura C. A. L. McKellar S. Swan D. G. Okan E. Shiels B. R. (2006). Infection of bovine cells by the protozoan parasite *Theileria annulata* modulates expression of the ISGylation system. *Cell. Microbiol.* 8 276–288. 10.1111/j.1462-5822.2005.00620.x 16441438

[B86] Ozaki T. Nakagawara A. (2011). P53: the attractive tumor suppressor in the cancer research field. *J. Biomed. Biotechnol.* 2011 1–13. 10.1155/2011/603925 21188172 PMC3004423

[B87] Pain A. Renauld H. Berriman M. Murphy L. Yeats C. A. Weir W. (2005). Genome of the host-cell transforming parasite *Theileria annulata* compared with *T. parva*. *Science* 309 131–133. 10.1126/science.1110418 15994557

[B88] Pellé K. G. Jiang R. H. Y. Mantel P.-Y. Xiao Y.-P. Hjelmqvist D. Gallego-Lopez G. M. (2015). Shared elements of host-targeting pathways among apicomplexan parasites of differing lifestyles. *Cell. Microbiol.* 17 1618–1639. 10.1111/cmi.12460 25996544

[B89] Petronczki M. Lénárt P. Peters J.-M. (2008). Polo on the Rise—from mitotic entry to cytokinesis with Plk1. *Dev. Cell* 14 646–659. 10.1016/j.devcel.2008.04.014 18477449

[B90] Prado M. Eickel N. De Niz M. Heitmann A. Agop-Nersesian C. Wacker R. (2015). Long-term live imaging reveals cytosolic immune responses of host hepatocytes against *Plasmodium* infection and parasite escape mechanisms. *Autophagy* 11 1561–1579. 10.1080/15548627.2015.1067361 26208778 PMC4590598

[B91] Repnik U. Gangopadhyay P. Bietz S. Przyborski M. Griffiths G. Lingelbach K. (2015). The apicomplexan parasite babesia divergens internalizes Band 3, Glycophorin A and spectrin during invasion of human red blood cells. *Cell. Microbiol.* 17 1052–1068. 10.1111/cmi.12422 25628009

[B92] Ritchie K. J. Hahn C. S. Kim K. I. Yan M. Rosario D. Li L. (2004). Role of ISG15 protease UBP43 (USP18) in innate immunity to viral infection. *Nat. Med.* 10 1374–1378. 10.1038/nm1133 15531891

[B93] Rupp I. Sologub L. Williamson K. C. Scheuermayer M. Reininger L. Doerig C. (2011). Malaria parasites form filamentous cell-to-cell connections during reproduction in the mosquito midgut. *Cell Res.* 21 683–696. 10.1038/cr.2010.176 21173797 PMC3072464

[B94] Sangaré L. O. Yang N. Konstantinou E. K. Lu D. Mukhopadhyay D. Young L. H. (2019). *Toxoplasma* GRA15 activates the NF-K B pathway through interactions with TNF receptor-associated factors. *mBio* 10: e0808-19. 10.1128/mBio.00808-19 PMC6635525 31311877

[B95] Schmuckli-Maurer J. Kinnaird J. Pillai S. Hermann P. McKellar S. Weir W. (2010). Modulation of NF-Kappa B activation in *Theileria annulata*-infected cloned cell lines is associated with detection of parasite-dependent IKK signalosomes and disruption of the actin cytoskeleton. *Cell. Microbiol.* 12 158–173. 10.1111/j.1462-5822.2009.01386.x 19804486

[B96] Seitzer U. Gerber S. Beyer D. Dobschanski J. Kullmann B. Haller D. (2010). Schizonts of *Theileria annulata* interact with the microtubuli network of their host cell via the membrane protein TaSP. *Parasitol. Res.* 106 1085–1102. 10.1007/s00436-010-1747-8 20162433

[B97] Shaw M. K. (1991). The entry of *Theileria parva* sporozoites into bovine lymphocytes: evidence for MHC Class I involvement. *J. Cell Biol.* 113 87–101. 10.1083/jcb.113.1.87 1901066 PMC2288915

[B98] Shaw M. K. (1997). The same but different: the biology of theileria sporozoite entry into bovine cells. *Intern. J. Parasitol.* 27 457–474. 10.1016/s0020-7519(97)00015-5 9193940

[B99] Shaw M. K. (2002). “*Theileria* development and host cell invasion,” in *Theileria, World Class Parasites*, eds Dobbelaere D. A. E. McKeever D. J. (Boston, MA: Springer), 1–22. 10.1007/978-1-4615-0903-5_1

[B100] Shaw M. K. Tilney L. G. (1995). The Entry of *Theileria parva* merozoites into bovine erythrocytes occurs by a process similar to sporozoite invasion of Lymphocytes. *Parasitology* 111 455–461. 10.1017/s0031182000065951 11023409

[B101] Shaw M. K. Tilney L. G. Musoke A. J. Teale A. J. (1995). MHC Class I molecules are an essential cell surface component involved in *Theileria parva* Sporozoite binding to bovine lymphocytes. *J. Cell Sci.* 108 1587–1596. 7615677 10.1242/jcs.108.4.1587

[B102] Shiels B. Langsley G. Weir W. Pain A. McKellar S. Dobbelaere D. (2006). Alteration of host cell phenotype by *Theileria annulata* and *Theileria parva*: mining for manipulators in the parasite genomes. *Intern. J. Parasitol.* 36 9–21. 10.1016/j.ijpara.2005.09.002 16221473

[B103] Shiels B. R. McKellar S. Katzer F. Lyons K. Kinnaird J. Ward C. (2004). A *Theileria annulata* DNA binding protein localized to the host cell nucleus alters the phenotype of a bovine macrophage cell line. *Eukaryot. Cell* 3 495–505. 10.1128/ec.3.2.495-505.2004 15075278 PMC387639

[B104] Sinai A. P. Webster P. Joiner K. A. (1997). Association of host cell endoplasmic reticulum and mitochondria with the *Toxoplasma gondii* parasitophorous vacuole membrane: a high affinity interaction. *J. Cell Sci.* 110(Pt 17), 2117–2128. 9378762 10.1242/jcs.110.17.2117

[B105] Skilton R. A. Bishop R. P. Wells C. W. Spooner P. R. Gobright E. Nkonge C. (1998). Cloning and characterization of a 150 KDa microsphere antigen of *Theileria parva* that is immunologically cross-reactive with the polymorphic immunodominant molecule (PIM). *Parasitology* 117 321–330. 10.1017/s0031182098003163 9820853

[B106] Speer C. A. Tilley M. Temple M. E. Blixt J. A. Dubey J. P. White M. W. (1995). Sporozoites of *Toxoplasma gondii* lack Dense-Granule protein GRA3 and form a unique parasitophorous vacuole. *Mol. Biochem. Parasitol.* 75 75–86. 10.1016/0166-6851(95)02515-4 8720177

[B107] Spooner R. L. Innes E. A. Glass E. J. Brown C. G. D. (1989). *Theileria annulata* and *Theileria parva* infect and transform different mononuclear cells. *Immunology* 66 284–288. 2784413 PMC1385101

[B108] Stagg D. A. Dolan T. T. Leitch B. L. Young A. S. (1981). The initial stages of infection of cattle cells with *Theileria parva* sporozoites in vitro. *Parasitology* 83 191–197. 10.1017/s0031182000050150 6791118

[B109] Sultan A. A. Thathy V. Frevert U. Robson K. J. H. Crisanti A. Nussenzweig V. (1997). TRAP is necessary for gliding motility and infectivity of *Plasmodium* sporozoites. *Cell* 90 511–522. 10.1016/S0092-8674(00)80511-5 9267031

[B110] Swan D. G. Phillips K. Tait A. Shiels B. R. (1999). Evidence for localisation of a *Theileria* parasite AT hook DNA-binding protein to the nucleus of immortalised bovine host cells. *Mol. Biochem. Parasitol.* 101 117–129. 10.1016/s0166-6851(99)00064-x 10413048

[B111] Swan D. G. Stadler L. Okan E. Hoffs M. Katzer F. Kinnaird J. (2003). TashHN, a *Theileria annulata* encoded protein transported to the host nucleus displays an association with attenuation of parasite differentiation. *Cell. Microbiol.* 5 947–956. 10.1046/j.1462-5822.2003.00340.x 14641179

[B112] Swan D. G. Stern R. McKellar S. Phillips K. Oura C. A. L. Karagenc T. I. (2001). Characterisation of a cluster of genes encoding *Theileria annulata* AT hook DNA-binding proteins and evidence for localisation to the host cell nucleus. *J. Cell Sci.* 114 2747–2754. 11683409 10.1242/jcs.114.15.2747

[B113] Tajeri S. Langsley G. (2021). Theileria secretes proteins to subvert its host leukocyte. *Biol. Cell* 113 220–233. 10.1111/boc.202000096 33314227

[B114] Thieleke-Matos C. Lopes da Silva M. Cabrita-Santos L. Portal M. D. Rodrigues I. P. Zuzarte-Luis V. (2016). Host cell autophagy contributes to *Plasmodium* liver development. *Cell. Microbiol.* 18 437–450. 10.1111/cmi.12524 26399761

[B115] Toye P. Musoke A. Naessens J. (2014). Role of the polymorphic immunodominant molecule in entry of *Theileria parva* sporozoites into bovine lymphocytes. *Infect. Immun.* 82 1786–1792. 10.1128/iai.01029-13 24549329 PMC3993433

[B116] Toye P. G. Goddeeris B. M. Iams K. Musoke A. J. Morrison W. I. (1991). Characterization of a polymorphic immunodominant molecule in sporozoites and schizonts of *Theileria parva*. *Parasite Immunol.* 13 49–62. 10.1111/j.1365-3024.1991.tb00262.x 1901640

[B117] Tretina K. Gotia H. T. Mann D. J. Silva J. C. (2015). *Theileria*-transformed bovine leukocytes have cancer hallmarks. *Trends Parasitol.* 31 306–314. 10.1016/j.pt.2015.04.001 25951781

[B118] Unlu A. H. Tajeri S. Bilgic H. B. Eren H. Karagenc T. Langsley G. (2018). The secreted *Theileria annulata* Ta9 protein contributes to activation of the AP-1 transcription factor. *PLoS One* 13:e0196875. 10.1371/journal.pone.0196875 29738531 PMC5940210

[B119] Villares M. Berthelet J. Weitzman J. B. (2020). The clever strategies used by intracellular parasites to hijack host gene expression. *Semin. Immunopathol.* 42 215–226. 10.1007/s00281-020-00779-z 32002610

[B120] von Schubert C. Xue G. Schmuckli-Maurer J. Woods K. L. Nigg E. A. Dobbelaere D. A. E. (2010). The transforming parasite *Theileria* Co-Opts host cell mitotic and central spindles to persist in continuously dividing cells. *PLoS Biol.* 8:e1000499. 10.1371/journal.pbio.1000499 20927361 PMC2946958

[B121] Walker M. E. Hjort E. E. Smith S. S. Tripathi A. Hornick J. E. Hinchcliffe E. H. (2008). *Toxoplasma gondii* actively remodels the microtubule network in host cells. *Microb. Infect.* 10 1440–1449. 10.1016/j.micinf.2008.08.014 18983931 PMC2765197

[B122] Wang H.-J. Hsieh Y.-J. Cheng W.-C. Lin C.-P. Lin Y.-S. Yang S.-F. (2014). JMJD5 regulates PKM2 nuclear translocation and reprograms HIF-1 -mediated glucose metabolism. *Proc. Natl. Acad. Sci. U.S.A.* 111 279–284. 10.1073/pnas.1311249111 24344305 PMC3890888

[B123] Watts J. G. Playford M. C. Hickey K. L. (2016). *Theileria orientalis*: a review. *New Zeal. Vet. J.* 64 3–9. 10.1080/00480169.2015.1064792 26143684

[B124] Webster P. Dobbelaere D. A. Fawcett D. W. (1985). The entry of sporozoites of *Theileria parva* into bovine lymphocytes in vitro. Immunoelectron microscopic observations. *Eur. J. Cell Biol.* 36 157–162. 3922761

[B125] Weir W. Sunter J. Chaussepied M. Skilton R. Tait A. de Villiers E. P. (2009). Highly syntenic and yet divergent: a tale of two theilerias. *Infect. Genet. Evol.* 9 453–461. 10.1016/j.meegid.2009.01.002 19460310

[B126] Wiens O. Xia D. von Schubert C. Wastling J. M. Dirk A. E. D. Heussler V. T. (2014). Cell Cycle-dependent phosphorylation of *Theileria annulata* schizont surface proteins. *PLoS One* 9:e103821. 10.1371/journal.pone.0103821 25077614 PMC4117643

[B127] Williamson S. Tait A. Brown D. Walker A. Beck P. Shiels B. (1989). *Theileria annulata* sporozoite surface antigen expressed in *Escherichia Coli* elicits neutralizing antibody. *Proc. Natl. Acad. Sci. U.S.A.* 86 4639–4643. 10.1073/pnas.86.12.4639 2499888 PMC287326

[B128] Woods K. von Schubert C. Dobbelaere D. (2013). “Hijacking of host cell signaling by *Theileria*,” in *Protein Phosphorylation in Parasites*, eds Doerig C. Späth G. Wiese M. (Hoboken, NJ: Wiley), 179–198. 10.1002/9783527675401.ch09

[B129] Woods K. L. Theiler R. Mühlemann M. Segiser A. Huber S. Ansari H. R. (2013). Recruitment of EB1, a master regulator of microtubule dynamics, to the surface of the *Theileria annulata* Schizont. *PLoS Pathog.* 9:e1003346. 10.1371/journal.ppat.1003346 23675298 PMC3649978

[B130] World Malaria Report (2020). Available online at: https://www.who.int/publications/i/item/9789240015791 (accessed January 15, 2021).

[B131] Xue G. von Schubert C. Hermann P. Peyer M. Maushagen R. Schmuckli-Maurer J. (2010). Characterisation of Gp34, a GPI-anchored protein expressed by schizonts of *Theileria parva* and *T. annulata*. *Mol. Biochem. Parasitol.* 172 113–120. 10.1016/j.molbiopara.2010.03.018 20381541 PMC2880791

[B132] Yabsley M. J. Shock B. C. (2013). Natural history of zoonotic babesia: role of wildlife reservoirs. *Intern. J. Parasitol.* 2 18–31. 10.1016/j.ijppaw.2012.11.003 24533312 PMC3862492

